# Clinical and socio-demographic factors associated with dental extractions in a clinical sample

**DOI:** 10.1590/0103-6440202305355

**Published:** 2023-12-22

**Authors:** Karla Karen Bernal-Sánchez, Edith Lara-Carrillo, Ulises Velázquez-Enriquez, Juan Fernando Casanova-Rosado, Alejandro José Casanova-Rosado, Adriana Alejandra Morales-Valenzuela, Sonia Márquez-Rodríguez, Carlo Eduardo Medina-Solís, Gerardo Maupomé

**Affiliations:** 1 Center for Advanced Studies and Research on Dentistry Dr. Keisaburo Miyata, School of Dentistry, Autonomous University of the State of Mexico, Toluca, Mexico; 2 School of Dentistry, Autonomous University of Campeche, Campeche, Mexico; 3 School of Dentistry, Autonomous University of the State of Mexico, Toluca, Mexico; 4 Academic Area of Dentistry, Health Sciences Institute, Autonomous University of the State of Hidalgo, Pachuca, Mexico; 5 Richard M. Fairbanks School of Public Health, Indiana University/Purdue University, Indianapolis, USA

**Keywords:** Oral health, dental extractions, dental caries, periodontal disease

## Abstract

The objective of the present study was to identify the reasons for dental extractions in patients seeking dental care in a university dental clinic in Mexico. This is a cross-sectional study that assessed 284 consecutive patients at the School of Dentistry, Autonomous University of the State of Mexico between August 2017 and November 2018. In total, 505 extractions were performed. The dependent variable was the reason for extraction: 0) dental caries and ensuing sequels (reference category); 1) periodontal disease and ensuing sequels; and 2) other reasons. Sociodemographic, socioeconomic, and clinical variables were included as independent variables. The analysis was done with multinomial logistic regression (Stata 14.0). Out of all extractions, 63.6% (n=321) were due to dental caries and ensuing sequels; 22.0% (n=111) were due to periodontal disease and ensuing sequels; 5.3% (n=27) endodontic failure; 5.1% (n=26) prosthetic indications; 1.6% (n=8) orthodontic indications; and the rest (2.4%) were due to other reasons. In the multivariate model extractions due to periodontal disease vs dental caries were associated with occasionally smoking tobacco (Odds Ratio, OR=3.90) or daily tobacco use (OR=3.19); the tooth to be extracted having been previously restored (OR=2.35); extracted anterior as opposed to posterior teeth (OR =2.63); and patients with multiple extractions (OR=2.68). In the case of extractions due to “other reasons”, no variable was significant. Dental caries and periodontal disease were the main reasons for dental extraction in this sample. Several variables, mostly clinical, were associated with extractions for periodontal reasons.

## Resumo

O objetivo do presente estudo foi identificar os motivos das extrações dentárias em pacientes que procuram atendimento odontológico em uma clínica odontológica universitária no México. Este é um estudo transversal que avaliou 284 pacientes consecutivos na Faculdade de Odontologia da Universidad Autónoma del Estado de México entre agosto de 2017 e novembro de 2018. No total, foram realizadas 505 extrações. A variável dependente foi o motivo da extração: 0) cárie dentária e sequelas subsequentes (categoria de referência); 1) doença periodontal e sequelas subsequentes; e 2) outros motivos. Variáveis sociodemográficas, socioeconômicas e clínicas foram incluídas como variáveis independentes. A análise foi feita com regressão logística multinomial (Stata 14.0). De todas as extrações, 63,6% (n=321) foram devidas a cárie dentária e sequelas subsequentes; 22,0% (n=111) foram devidas a doença periodontal e sequelas subsequentes; 5,3% (n=27) a insucesso endodôntico; 5,1% (n=26) a indicações protéticas; 1,6% (n=8) a indicações ortodônticas; e o restante (2,4%) foi devido a outros motivos. No modelo multivariado, as extrações por doença periodontal vs. cárie dentária foram associadas ao tabagismo ocasional (Odds Ratio, OR=3,90) ou ao uso diário de tabaco (OR=3,19); ao fato de o dente a ser extraído ter sido restaurado anteriormente (OR=2,35); à extração de dentes anteriores em vez de posteriores (OR=2,63); e a pacientes com extrações múltiplas (OR=2,68). No caso de extrações devido a "outros motivos", nenhuma variável foi significativa. A cárie dentária e a doença periodontal foram os principais motivos de extração dentária nessa amostra. Diversas variáveis, principalmente clínicas, foram associadas às extrações por motivos periodontais.

## Introduction

Oral health is a critical component of overall health and well-being. Oral disorders are an important public health issue that affects a substantial portion of the global population. Such a considerable burden of disease is caused by untreated dental caries in both dentitions, severe periodontitis, and multiple tooth loss. Caries and periodontitis are the most prevalent oral diseases and, when not treated, they can have psychosocial and physiologic impacts on individuals, potentially diminishing their quality of life. The burden of caries and periodontitis disease leading to tooth loss is significant^1^. Oral diseases continue to be a serious public health challenge on a global scale because there has been little improvement in oral health over the past three decades. Between 1990 and 2015, the burden of oral disorders grew significantly as a result of demographic changes, such as population expansion and aging. Scientific evidence showed that the number of persons with untreated oral conditions went from 2.5 billion in 1990 to 3.5 billion in 2015^2^.

Various reasons for dental extraction in adults have been reported around the world^3^, including Mexico^4^ ^,^ ^5^. Overall, caries and periodontal disease are the main reasons for dental extractions. Other reasons reported are prosthetic and orthodontic issues, dental trauma, tooth impaction (mainly third molars), endodontic treatment failure, medical indications, iatrogenesis, and apical pathoses^3^. There are other non-dental factors associated with dental extraction such as chronic diseases, as in the case of more dental extractions in patients with diabetes and tobacco users^6^ ^,^ ^7^. Tooth loss is a clinical phenomenon modified by patients’ attitudes, dentists’ clinical practices, doctor-patient relationships, availability and accessibility of dental services, as well as prevailing philosophies in dental care^8^ ^,^ ^9^. According to this landscape, it is important to identify local risk factors to screen subjects at risk of losing teeth as well as to help inform clinical decisions. Severe tooth loss was the 36th most prevalent condition worldwide, hence representing a significant healthcare, social, and economic burden^1^.

### Dental health care system in Mexico

The country's health care system is a complex array of multiple providers; it offers dental care services to the general public through a combination of employment-linked dental insurance paid for by third parties and out-of-pocket dental treatment, primarily at the point of service. Many dental care services are almost solely supplied at a fee-per-item per visit, paid by the patient; the few publicly-funded providers do not offer specialist care. The Mexican Army and Navy and the National Oil Company (PEMEX) dental insurance systems offer more complete clinical options with greater coverage. In 2003, the Mexican government set up *Seguro Popular*, a public health care program free to the poorest population sectors; it offered partial dental coverage funded by federal and state governments and to a lesser extent by households. The program was discontinued in 2020. Finally, many public and private dental schools offer dental services delivered by students at much lower costs than the private dental care market^10^.

The objective of the present study was to identify the reasons for dental extractions in patients seeking dental care in a university dental clinic in Mexico.

## Materials and methods

### Design, population, and study sample

This is a cross-sectional study that assessed 284 consecutive patients who visited dental clinics in the School of Dentistry, Autonomous University of the State of Mexico (UAEMex) between August 2017 and November 2018. Clinics offer services to the general population; most patients have middle to low socioeconomic status.

The sample size was calculated using a confidence level of 95%, accuracy of 4%, proportion to estimate 43%^4^, and estimated loss proportion of 15%. The sample size was 497. Sampling was systematic, not probabilistic, and included all consecutive patients who needed extraction of one or more teeth and met inclusion and exclusion criteria.

Inclusion criteria were patients of either sex, 12 years of age and older, needing extraction of a permanent tooth, with or without any general health condition, and who agreed to participate and signed the informed consent form. Exclusion criteria were cases needing extraction of supernumerary teeth retained deciduous teeth, or refusal to take part in the study.

### Data collection and variables and data gathering

Clinical and radiographic exams were conducted by dental students under the supervision of dental faculty who helped them with diagnosis and treatment plans. Calibrated senior dental students filled out the data forms after securing informed consent from the patient; data collection preceded the clinical service. The dependent variable was the main reason for extraction, which was classified as follows: 1) dental caries and ensuing sequels; 2) failure of endodontic treatment; 3) periodontal disease and ensuing sequels; 4) prosthetic reasons; 5) orthodontic reasons; 6) dental trauma; 7) occlusion problems; and 8) other reasons.

A clinical exam and a questionnaire were administered to patients to collect data on independent variables and self-reported systemic conditions. Independent variables were age (from 13 to 84 years); sex (1: female; 2: male); maximum level of schooling (0 - 18 years of study); any restoration present in the tooth scheduled for extraction (0: no restorations; 1: inlay; 2: full crown or part of a bridge; 3: resin or amalgam); patient requiring multiple extractions during the study (1: One extraction; 2: More than one extraction); having systemic disease (0: none; 1: diabetes; 2: hypertension); and tobacco use at any point in life (0: no; 1: yes); current tobacco use (0: Never or not currently; 1: Occasional; 2: Yes, every day [at least one cigarette a day]); location for extraction in terms of dental arch (0: Maxilla; 1: Mandible), type of tooth targeted for extraction (0: Anterior, 1: Posterior). All patients were treated in a dental chair under local anesthesia.

### Statistical analysis

Statistical analysis was carried out with Stata 14 in three steps: univariate, bivariate, and multivariate. In the univariate analysis, a description of continuous variables included central tendency and dispersion measures; for categorical variables, frequencies and percentages were obtained. In the bivariate and multivariate analyses, a multinomial logistic regression model was utilized since the dependent variable was categorized for analysis as 0) dental caries and ensuing sequels; 1) periodontal disease and ensuing sequels; and 2) other reasons. The strength of association between dependent and independent variables is expressed as odds ratios (OR) with 95% confidence intervals (95% CI). To build the multivariate model, variables with a value of p<0.25 in the bivariate analyses were taken into consideration^11^. In bivariate and multivariate analyses, confidence intervals were calculated with robust standard errors, which enabled valid estimations even in the case of correlation inside the groups (mouth). This strategy was adopted because there was a correlation between tooth statuses in patients who needed more than one extraction^12^.

### Ethical issues

The research protocol was approved by the Committee on Ethical Research (CEICIEAO - 004). The indications from the Declaration of Helsinki and the General Law on Research on Health Care in Mexico were followed.

## Results

The study comprised 505 extractions performed on 284 patients (an average of 2.75 ± 1.92 extractions per patient). In total, 186 patients (65.5%) were women; the overall mean age was 46.81±16.11 years; the average schooling years was 9.0. Most extracted teeth (85.7%) did not have restorations. Many patients (66.9%) required multiple extractions. Systemic disease was self-reported by 23.6% of the patients, with diabetes and hypertension being the most prevalent. Smoking was self-reported by 28.5% of patients (Table 1).

Dental caries and its sequels were the most frequent reasons for dental extractions (n=321, 63.6%), followed by periodontal disease and its sequels (n=111, 22.0%). Endodontic treatment failure was the third reason with 27 extractions (5.3%) followed by prosthetic indications (n= 26, 5.1%), orthodontic indications (n=8, 1.6%), dental trauma (n=3, 0.6%), occlusal problems (n=4, 0.8%) and other reasons (n=5, 1.0%). Lower first molars (36 and 46) were the most frequently extracted teeth. Teeth with the highest prevalence of extraction were first molars (21.6%), followed by second molars (14.2%). The teeth with the lowest prevalence were lateral incisors (8.9%). The most commonly extracted teeth were the right and left lower third molars (5.8%). Lower teeth had a higher prevalence of extraction (50.8%) than upper teeth (Table 4).

Table 2 shows the multinomial logistic regression bivariate analysis, dental caries and its sequels vs. periodontal disease and its sequels. The variables associated (p<0.05) were age, patient requiring multiple extractions during the study interval, type of teeth, and tobacco use. When dental caries and its sequels vs. other reasons were analyzed, associated variables (p<0.05) were tobacco use at any point in life and current tobacco use.

Table 3 shows the results of the multinomial logistic regression multivariate analysis. Tobacco use increased the likelihood of extraction due to periodontal disease but not due to caries [occasional use (OR=3.90, 95% CI = 1.17 - 13.04) and tobacco daily use (OR=3.19, 95% CI = 1.15 - 8.90)]. Restored teeth were 2.35 times (95% CI = 1.03 - 5.38) more likely to be extracted due to periodontal disease than dental caries. Anterior teeth were more likely to be extracted due to periodontal disease than dental caries (OR=2.63, 95% CI = 1.35 - 5.00). Patients with multiple extractions were more likely to have teeth extracted because of periodontal disease than caries (OR=2.68, 95% CI = 1.25 - 5.72). In the case of other categories, such as for other reasons, vs. caries and its sequels, no variable was significant.

**Table 1:** Sociodemographic and dental/medical characteristics of the study participants

Variables	Mean ± SD	Limits
Age (years)	46.81± 16.11	13-84
Schooling level (years)	9.0 ± 4.4	0-18
Number of extractions during the study	2.75 ± 1.92	1-9
	n	%
Sex		
Female		
Male	18698	65.534.5
Dental arch		
Maxilla	255	50.4
Mandible	250	49.6
Group of teeth		
Anterior	152	30.0
Posterior	353	70.0
Restorations in teeth to be extracted		
No	433	85.8
Partial crown or inlay	9	1.8
Full crown or bridge	15	2.9
Other restoration (resin or amalgam)	48	9.5
Multiple extractions during the study		
One extraction	167	33.1
More than one extraction	338	66.9
Systemic disease (any)		
No	217	76.4
Yes	67	23.6
Diabetes		
No	251	88.4
Yes	33	11.6
Hypertension		
No	249	87.7
Yes	35	12.3
Tobacco use		
No	203	71.5
Yes	81	28.5
Current tobacco use		
No	237	83.5
Yes, occasional	19	6.7
Yes, daily (at least one cigarette)	28	9.8
Reasons for extraction		
Caries and its sequels	321	63.6
Periodontal disease and its sequels	111	22.0
Other reasons	73	14.4

**Table 2:** Association between reasons for extraction of permanent teeth and independent variables. Part 1

	Periodontal disease and its sequels	Other reasons
Variable	OR (95% CI);	OR (95% CI)
Age (years)	1.03 (1.00 - 1.05)‡	1.00 (0.97 - 1.03)^n/s^
Schooling level (years)	0.95 (0.88 - 1.04)^n/s^	1.05 (0.95 - 1.16)^n/s^
Sex		
Male	1*	1*
Female	1.53 (0.72 - 3.24)^n/s^	1.50 (0.58 - 3.75)^n/s^
Dental arch		
Maxilla	1*	1*
Mandible	1.40 (0.73 - 2.58)^n/s^	0.95 (0.42 - 2.12)^n/s^
Group of teeth		
Anterior	1*	1*
Posterior	0.29 (0.16 - 0.54)¶	0.88 (0.40 - 1.96)^n/s^
Tooth restoration		
Restored	1*	1*
Unrestored	1.30 (0.60 - 2.84)^n/s^	2.03 (0.86 - 4.80)^n/s^
Multiple extractions during the study		
One extraction	1*	1*
More than one extraction	3.34 (1.70 - 6.57)¶	0.91 (0.43 - 1.94)^n/s^
Diabetes		
No	1*	1*
Yes	2.13 (0.85 - 5.33)^n/s^	1.60 (0.31 - 8.30)^n/s^
Hypertension		
No	1*	1*
Yes	2.09 (0.81 - 5.37)^n/s^	0.50 (0.15 - 1.70)^n/s^
Tobacco use at any point in life		
No	1*	1*
Yes	1.60 (0.75 - 3.43)^n/s^	0.40 (0.17 - 0.92)†
Current tobacco use		
Never or not currently	1*	1*
Occasionally or daily	1.7 (1.09 - 2.86)¶	0.54 (0.30 - 0.96)†

*Reference category

Crude multinomial logistic regression analysis

The reference group for each variable was extraction due to caries.

Confidence intervals were calculated with robust standard errors per mouth cluster.

**Table 3:** Association between reasons for extraction of permanent teeth and independent variables. Part 2

	Periodontal disease and its sequels	Other reasons
Variable	OR (95% CI)	OR (95% CI)
Current cigarette smoking		
No	1*	1*
Occasionally	3.90 (1.17 - 13.04)†	1.65 (0.56 - 4.85)^n/s^
Yes, daily (at least 1 cigarette per day)	3.19 (1.15 - 8.90)†	not calculated
Tooth restoration		
Previously unrestored	1*	1*
Previously restored	2.35 (1.03 - 5.38)†	2.13 (0.88 - 5.19)^n/s^
Group of teeth		
Posterior	1*	1*
Anterior	2.63 (1.35 - 5.00)‡	1.35 (0.65 - 2.85)^n/s^
Multiple extractions during the study		
One extraction		
More than one extraction	2.68 (1.25 - 5.72)†	1.03 (0.48 - 2.22)^n/s^

*Reference category

Multinomial logistic regression multivariate analysis.

The reference group for each variable was extraction due to caries.

Note: Model adjusted for the variables contained in the table in addition to age and sex. Confidence intervals were calculated with robust standard errors per mouth cluster.

n/s = not significant, † <0.05, ‡ <0.01

**Table 4:** Prevalence of extraction per tooth type

Tooth type	n	Percentage
18	8	1.6
17	11	2.1
16	9	1.8
15	15	3.0
14	21	4.1
13	25	5.0
12	10	2.0
11	14	2.8
21	19	3.8
22	14	2.8
23	15	3.0
24	21	4.1
25	17	3.3
26	25	5.0
27	18	3.5
28	13	3.0
38	16	3.1
37	13	2.5
36	11	2.1
35	6	1.1
34	8	1.5
33	29	5.8
32	21	4.1
31	11	2.1
41	15	3.0
42	8	1.5
43	13	2.6
44	18	3.6
45	19	3.8
46	29	5.8
47	19	3.8
48	14	2.8
Total	505	100

## Discussion

The present study identified reasons for the extraction of permanent teeth in individuals 12 years of age and older in a clinical sample. Worldwide, dental caries, periodontitis, and severe tooth loss are major oral diseases. They are a public health problem because of their high prevalence and incidence, which have increased in recent years^1^ ^,^ ^2^ ^,^ ^13^. Unfortunately, dental health has not substantially improved over the last three decades, and oral conditions are still an important challenge for health care systems in many countries: the number of people with untreated oral conditions increased from 2.5 billion in 1990 to 3.5 billion in 2015^2^. Several observational studies reported that dental caries and periodontal disease are the most common indications for extraction of permanent teeth, although the relative importance could vary between studies, with noticeable effects for age (reasons for tooth loss differ between age groups), year when research was published (reasons for tooth loss change over time) and specific population group (reasons for tooth loss differ according to socioeconomic status)^13^ ^,^ ^14^. It is important to distinguish that the main reasons for extraction we observed (even if with slight changes in order) are similar to those reported in previous reports for Mexico^4^ ^,^ ^5^ and other parts of the world^3^. The costs of dental restorations are generally high and thus may be an important component in a decision to extract a tooth^15^, opting for an extraction instead of a conservative treatment option such as restorations. Payment for dental care in the clinics used in the study to restore a tooth may be up to 13 times higher than extracting it.

Multiple factors must be taken into account to better situate the phenomenon of extractions. Recent research has examined the strength of the association between tobacco use and tooth loss attributed to periodontal disease^16^. Physiologically, tobacco use causes a decline in blood flow, leading to cell and microbiological changes in periodontal tissues^17^. Such a shift increases the periodontopathogenic bacteria population, inflammatory response, vasoconstriction, osteoclastic activity, and tissue ischemia; all of these affect the periodontium’s repair response^18^. Effects of tobacco on saliva pH suggest a clear difference between smokers and nonsmokers, with the former having diminished buffer capacity. Any decline in salivary flow promotes a larger accumulation of dental plaque and calculus, which may be damaging to periodontal tissues^19^. Tobacco cessation is generally beneficial to teeth; the existing evidence, even if limited, shows that smoking cessation may result in additional benefits to outcomes of nonsurgical periodontal treatment^20^.

We found that teeth with restorations were more likely to be scheduled for extraction, with the stated reasons for extraction being associated with periodontal disease. It may also be possible that this increased risk of extraction is a consequence of treatment decisions by dentists, ascribable to, e.g., defective margins in fillings. The conventional wisdom is that such imperfections promote the accumulation of dental plaque and eventually lead to attachment loss and impacts on the alveolar bone^21^ ^,^ ^22^. Alternatively, it may be simply that clinicians attach lower value to preserving restored teeth - in particular, if the restorations have perceived or actual shortcomings^23^.

Extractions due to periodontal reasons were significantly higher for anterior teeth in this population group. Montadon et al.^24^ found that extractions of anterior teeth increase with age, whereas extractions in posterior teeth are greater in younger populations. It is unclear whether those trends may result from higher caries experience among the young, compared to periodontal disease and its sequels among the older population. Disparate anatomic features between anterior and posterior teeth may also be a contributing factor^25^.

Results from the present study suggest implications for both health systems and health policy. Namely, the cost of care, whereby the extraction itself may be not particularly costly but subsequent prosthetic rehabilitation care is often costly. Again, out-of-pocket costs at the point of care are challenging for some patients. Such considerations and the limited array of clinical options covered by third-party insurance should be part of the landscape where impacts are evaluated. Additionally, differential patterns of tooth extractions emphasize the role of health disparities; whether they underlie reasons for extractions (e.g., caries in the lower socioeconomic status groups, orthodontic extractions in the better off patients) or not, health systems could ameliorate health inequities through assuring better access to clinical care independently of ability to pay at the point of care, implementing preventive programs (i.e., fluoride technologies), and ensuring that early interventions are available to high-risk population groups. In the larger scheme of things, such measures would have an impact on focusing the skillsets acquired during dental education, the distribution of dental care options in those areas not necessarily favored by private clinical care, and specific measures to alleviate dental conditions before an extraction is indicated.

The present study has some limitations we must take into account to place more accurately the value of findings. The first is related to design; as this study was cross-sectional, causal relations cannot be established. The population under study was restricted to patients actively seeking care at a dental clinic catering to the medium and low socioeconomic strata in the Mexican dental market; such a population is not representative of the overall dental market or the population in the country.

In conclusion, dental caries and periodontal disease (and sequels of both conditions) were the main reasons for dental extractions in this sample. In the final multivariate model, several variables -- mainly clinical -- were associated with extractions for periodontal reasons.
